# Transformation and outcome of nodular lymphocyte predominant Hodgkin lymphoma: a Finnish Nationwide population-based study

**DOI:** 10.1038/s41408-021-00586-1

**Published:** 2021-12-18

**Authors:** Ilja Kalashnikov, Tomas Tanskanen, Janne Pitkäniemi, Nea Malila, Sirkku Jyrkkiö, Sirpa Leppä

**Affiliations:** 1 Research Program Unit, Applied Tumor Genomics, Faculty of Medicine, University of Helsinki, Helsinki, Finland; 2 Department of Oncology, Helsinki University Hospital Comprehensive Cancer Center, Helsinki, Finland; 3 iCAN Digital Precision Cancer Medicine Flagship, Helsinki, Finland; 4 Finnish Cancer Registry, Institute for Statistical and Epidemiological Cancer Research, Helsinki, Finland; 5 Department of Public Health, School of Medicine, University of Helsinki, Helsinki, Finland; 6 Faculty of Social Sciences, University of Tampere, Tampere, Finland; 7 Department of Oncology and Radiotherapy, Turku University Hospital, Helsinki, Finland

**Keywords:** Epidemiology, B-cell lymphoma

## Abstract

Nodular lymphocyte predominant Hodgkin lymphoma (NLPHL) is a rare B-cell malignancy associated with excellent survival. However, some patients experience histological transformation into aggressive large B-cell lymphoma. Population-based data on transformation in patients with NLPHL is limited. We conducted a nationwide population-based study to estimate the risk of transformation and relative survival in patients diagnosed with NLPHL in Finland between 1995 and 2018. We identified a total of 453 patients (median age, 48 years; 76% males) with the incident NLPHL from the Finnish Cancer Registry. The cumulative incidence of transformation was 6.3% (95% CI, 4.2-9.6) at 10 years. After adjusting for sex, age and year of diagnosis, transformation was associated with a substantially increased risk of death (HR 8.55, 95% CI 4.49−16.3). Ten-year relative survival was 94% (95% CI, 89%‒100%). The patients diagnosed at a later calendar year had lower excess risk of death (HR, 0.38 per 10-year increase; 95% CI, 0.15‒0.98). We conclude that while the 10-year relative survival for the patients with NLPHL was excellent in this large population-based cohort for the entire study period, transformation resulted in a substantially increased mortality compared with the patients without transformation. Our results also suggest a reduction in excess mortality over time.

## Introduction

Worldwide, Hodgkin lymphoma (HL) accounted for an estimated 83 000 (0.4%) new cancer cases and 23 000 (0.2%) cancer deaths in 2020 [1]. While the age-standardized incidence remained rather stable, the age-standardized mortality and disability adjusted life-year rates have steadily decreased since the 1990s [1, 2], resulting in excellent survival rates. For example, between 2014 and 2018 in the Nordic countries, 5-year relative survival was over 85%. In Finland, approximately 180 new HL cases are diagnosed annually [2, 3].

Nodular lymphocyte predominant Hodgkin lymphoma (NLPHL) is a rare subtype of HL with unique clinicopathological features, and an estimated incidence of 1-3 per million per year, representing 5-15% of all new HL cases [4–7]. In contrast to classical HL (cHL), NLPHL mainly affects middle-aged males, clinical course of the disease is generally indolent, and most patients present with limited-stage disease at diagnosis. Also, there is a risk of late disease progression and histological transformation into large B-cell lymphoma [8, 9].

Population-based data on transformation in patients with NLPHL is limited since the majority of cancer registries do not capture transformation status. Therefore, the current literature is mainly comprised of retrospective single-center studies. The results regarding the impact of transformation on survival are also inconclusive [10–14].

In comparison to cHL, NLPHL patients have better survival, and in young adults, survival may even be comparable to the general population [15–17]. Excess mortality is low and largely associated with transformation to large B-cell lymphoma and treatment-related long-term effects, such as secondary malignant neoplasms and cardiovascular diseases [11, 18].

There are no randomized controlled trials, and only a few prospective studies on NLPHL have been conducted to guide treatment decisions. Historically, patients with NLPHL have been treated similarly to cHL. However, increasing evidence has accumulated during the last two decades to support the use of less aggressive therapeutic approaches [19]. Patients with limited-stage disease are usually managed with radiotherapy or combined modality treatment, whereas systemic treatment with combination chemotherapy is indicated for the patients with advanced-stage disease [19]. Since NLPHL cells express CD20, biological agents, such as rituximab, have emerged as an additional treatment option since the early 2000s [19].

We conducted a nationwide population-based study to estimate the risk of transformation to large B-cell lymphoma and relative survival in patients diagnosed with NLPHL in Finland in 1995−2018. We also compared mortality rates between patients with or without transformation.

## Methods

### Data sources

The Finnish Cancer Registry (FCR) is a statistical and epidemiological research institute responsible for registering nationwide data on all incident cancer diagnoses in Finland since 1953. Physicians, hospitals, and pathology and hematology laboratories are obliged to report cancer cases to the FCR without consent of the patient based on a special legislation. The reports include a unique personal identity code, which allows for linkage between national registries and reliable follow-up of cancer patients. Extensions, recurrences, metastases, or transformations of previously recorded primary cancers are reported to the FCR but are not registered as separate cases. Since 2007, the coding of cancer cases has followed the International Classification of Diseases for Oncology, 3rd Edition (ICD-O-3), which is consistent with the WHO Classification of Tumours of Haematopoietic and Lymphoid Tissues, 4th edition [4, 20]. In 1953–2006, cancer morphology was coded according to a slightly modified version of the Manual of Tumor Nomenclature and Coding and topography according to the International Classification of Diseases, 7th revision. In 2007, former codes were converted to ICD-O-3 [21, 22]. Information regarding vital status and place of residence of cancer patients is obtained continuously from the Population Information System maintained by the Digital and Population Data Services Agency. Causes of death for cancer patients are received from Statistics Finland once per year. The FCR has high coverage overall, and in a recent quality study, the completeness for HL was estimated at 93.4%, and 99.7% of HL cases were morphologically verified [23, 24].

### Patients

Patients diagnosed with incident NLPHL (ICD-O-3 morphology code 9659/3) in Finland in 1995–2018 were retrieved from the FCR database. Patient selection was restricted to those diagnosed as of 1995 to ensure a representative cohort considering that NLPHL was first recognized as a distinct entity and separated from the cHL subtypes in the revised European American lymphoma (REAL) classification of 1994 [25, 26]. A total of 485 patients were initially identified. The diagnosis of NLPHL and possible transformation were confirmed from the free-text part of the pathology reports. Clinical reports were used for additional confirmation. In unclear cases, additional information was requested from the pathology laboratories. We excluded 11 patients with incorrect registry entries or missing cancer notifications and 5 patients diagnosed with other lymphoma before NLPHL. Sixteen patients diagnosed concurrently with a combination of NLPHL and a large B-cell lymphoma, i.e., composite lymphoma, were also excluded from the study. Therefore, the study population consisted of 453 patients. None of the NLPHL diagnoses or transformations were registered by death certificate or autopsy only. Incident transformation was defined as the diagnosis of morphologically verified large B-cell lymphoma or high-grade B-cell lymphoma at least 3 months after the primary diagnosis of NLPHL.

The statistical underlying causes of death for deceased NLPHL patients were retrieved from Statistics Finland. Causes of death were determined according to the selection and application rules of the International Classification of Diseases (ICD-10) compiled by the World Health Organisation (WHO).

For all NLPHL patients, we collected data on sex; date of birth, diagnosis of NLPHL, and last follow-up; vital status at the end of follow-up; possible date of transformation; and cause of death. The study cohort was followed until December 31, 2018. One patient was lost to follow-up before the end of the study period. General population mortality rates of Finland were obtained from Statistics Finland. Cause of death was categorized into three groups: any lymphoma, secondary malignant neoplasm, or other cause.

The study was approved by the National Institute for Health and Welfare (Dnro THL/1441/5.05.00/2019), Statistics Finland (Dnro TK-53-1172-19), and Helsinki University Hospital Institutional Review Board.

### Statistical analysis

The start of follow-up was defined as the date of NLPHL diagnosis. The Kaplan-Meier method was used to estimate overall survival. A competing risk method (Aalen-Johansen estimator) was used to estimate the cumulative incidence of transformation, with death due to other causes as the competing risk. Hazard ratios (HRs) for death from any cause and incident transformation were estimated using multivariable Cox regression models. Age at diagnosis was modeled as a linear continuous variable and year of diagnosis as a linear continuous or a categorical variable.

Relative survival was estimated using the Ederer II method with internal age-standardization (age groups: 0−14, 15−44, 45−54, 55−64, 65−74, and ≥75 years) and monthly intervals for collapsing the data [27]. Complete analysis was based on all person-time and deaths in 1995−2018. Period analyses were carried out for 1995−2006 and 2007−2018, with left-truncated data for the later period [28].

To estimate HRs for excess mortality, we used multivariable flexible parametric survival models [29]. Expected mortality was based on the general population mortality rates of Finland by 1-year age group, calendar year, and sex. The baseline excess hazard rate was modeled with 4 degrees of freedom [30].

When analyzing total and excess mortality, transformation was treated as a time-varying covariate. To allow for non-proportional hazards, we fit separate models that also included time from transformation. After splitting the time scale into monthly intervals, the time-dependent effect of transformation was modeled with a restricted cubic spline with 2 degrees of freedom.

The Wilcoxon rank-sum test was used to compare age at diagnosis between groups.

Statistical analyses were performed using R, version 4.0.2 (R Foundation for Statistical Computing, Vienna, Austria), with the packages survival 3.1-12, rstpm2 1.5.2, Epi 2.44, and popEpi 0.4.8.

## Results

### Patients

We identified 453 patients diagnosed with NLPHL in Finland between January 1995 and December 2018. Baseline characteristics of the patients, as well as age-specific rates of transformation and death, are shown in Table 1. The majority of the patients were males (76%). The median age at diagnosis was 48 years (interquartile range (IQR), 34−61; range, 5–87). Females were significantly older than males at the time of diagnosis (median age, 58 vs. 45 years; *p* < 0.001). The median follow-up time was 8.1 years (IQR, 3.4−14.1; range, 0‒24 years).

**Table 1:** Characteristics of patients diagnosed with nodular lymphocyte predominant Hodgkin lymphoma.

Age at diagnosis, years	No. of patients (%)	Male/female	Median year of diagnosis (IQR)	Median follow-up, years (IQR)	Follow-up time, person-years	No. of deaths (%)	Mortality rate (per 1000 person-years)	No. of transformations (%)	Transformation rate (per 1000 person-years)
0−14	12 (2.6)	12/0	2001 (1997−2013)	17.6 (6.4−20.5)	173	1 (1.3)	5.8	0 (0.0)	0.0
15−39	145 (32.0)	117/28	2009 (2001−2013)	9.9 (4.5−15.7)	1558	10 (12.8)	6.4	9 (36.0)	6.0
40−64	209 (46.1)	167/42	2009 (2002−2015)	8.8 (3.5−14.9)	1987	30 (38.5)	15.1	10 (40.0)	5.2
≥65	87 (19.2)	50/37	2012 (2004−2015)	4.1 (1.5−7.8)	464	37 (47.4)	79.8	6 (24.0)	13.1
All ages	453 (100.0)	346/107	2009 (2002−2015)	8.1 (3.4−14.1)	4182	78 (100.0)	18.6	25 (100.0)	6.2

### Risk of transformation

**Fig. 1:**
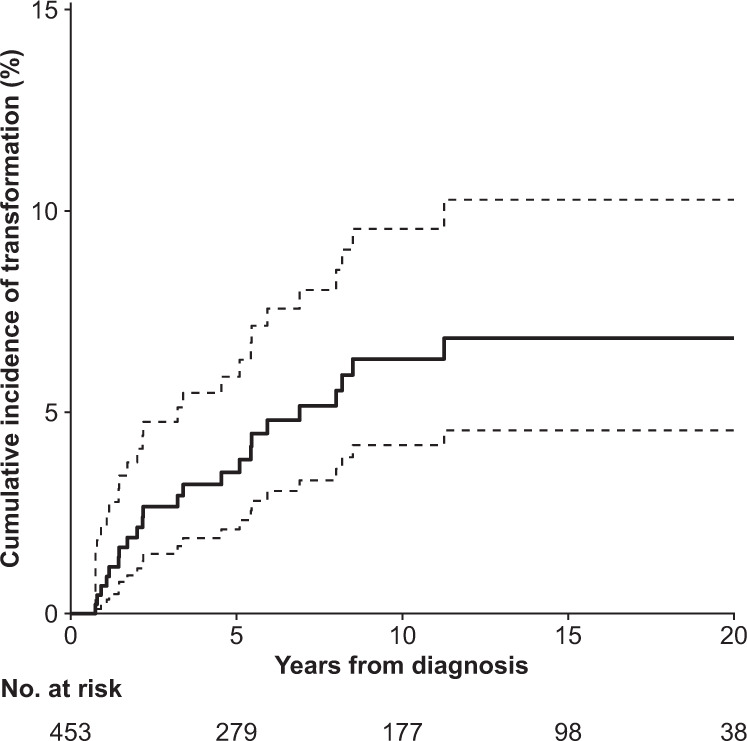
Cumulative incidence of transformation. The dashed lines indicate 95% confidence intervals.

Incident transformation occurred in 25 patients during 4059 person-years of follow-up (crude transformation rate, 6.2/1000). The cumulative incidence of transformation was 3.5% (95% CI, 2.1–5.9) at 5 years and 6.3% (95% CI, 4.2–9.6) at 10 years from diagnosis, reaching a plateau thereafter (Fig. 1). The majority of the transformations (22 of 25) occurred within 10 years from diagnosis; one occurred between 10–19 years and two after 20 years. Of the 25 transformations, 18 (72%) occurred in males. The median age at the time of transformation was 57 years (IQR, 40–69; range, 27–87). The risk of transformation was not significantly associated with age at diagnosis (HR, 1.22 per 10-year increase; 95% CI, 0.94–1.57; *p* = 0.13), sex (HR, 1.26 for females compared with males; 95% CI, 0.50–3.18; *p* = 0.6), or year of diagnosis (HR, 0.73 per 10-year increase; 95% CI, 0.37–1.43; *p* = 0.4). In addition, after adjustment for age at diagnosis and sex, we found no significant association with the period of diagnosis (HR, 0.66 for 2005‒2018 compared with 1995‒2004; 95% CI, 0.29‒1.53, *p* = 0.3). The histology at the time of transformation was diffuse large B-cell lymphoma NOS in 15 patients (60%) and T-cell/histiocyte rich large B-cell lymphoma in 10 patients (40%).

### Overall survival

There were 78 deaths during a follow-up of 4 182 person-years (crude mortality rate, 18.6/1 000). The overall survival probability was 85% (95% CI, 81%–89%) at 10 years and 65% (95% CI, 58%–74%) at 20 years from diagnosis.

Among the patients with transformation, 12 died during 123 years of follow-up after transformation. The crude mortality rate following incident transformation was 97.4/1000 compared with 16.3/1000 among patients without transformation.

**Fig. 2:**
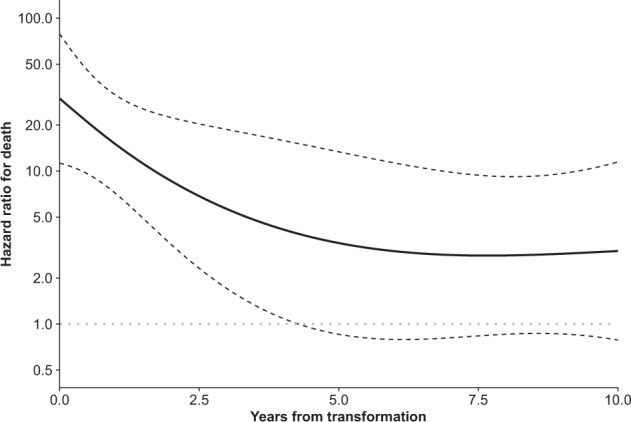
Hazard ratio for death (on a logarithmic scale on the y-axis) by the time from transformation. The dashed lines indicate 95% confidence intervals.

After adjusting for potential confounders (age at diagnosis, sex, and year of diagnosis), transformation was associated with a substantial increase in total mortality (HR, 8.55; 95% CI, 4.49–16.3; *p* < 0.001) (Table 2). The relative risk of death, as compared with the patients without transformation, appeared to be highest at the time of transformation (Fig. 2). The time-dependent HRs at 1, 5, and 10 years after transformation were 15.0 (95% CI, 7.16‒31.4), 3.38 (95% CI, 0.86‒13.4), and 3.00 (95% CI, 0.79–11.5), respectively.

**Table 2:** Hazard ratios for total and excess mortality in patients with nodular lymphocyte predominant Hodgkin lymphoma.

Characteristic	Total mortality			Excess mortality		
	HR^a^	95% CI^b^	*p*-value	HR^a^	95% CI^b^	*p*-value
Age at diagnosis (per 10-year increase)	2.09	1.74−2.52	<0.001	1.75	1.15−2.66	0.009
Sex						
Male	1.00	—	—	1.00	—	—
Female	0.71	0.42−1.22	0.2	0.89	0.29−2.77	0.8
Year of diagnosis (per 10-year increase)	0.52	0.34−0.80	0.003	0.38	0.15−0.98	0.046
Transformation						
Nontransformed	1.00	—	—	1.00	—	—
Transformed	8.55	4.49−16.3	<0.001	26.7	9.41−75.9	<0.001

^a^ HR, hazard ratio

^b^ CI, confidence interval

### Relative survival and excess risk of death

**Fig. 3:**
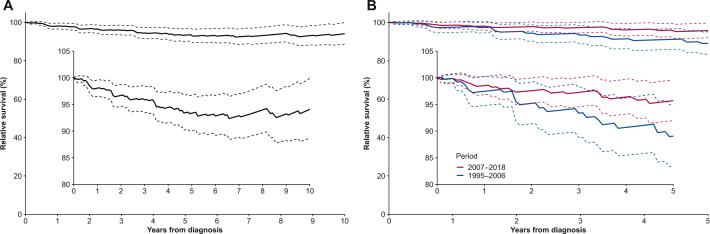
Relative survival estimates for patients diagnosed with NLPHL in Finland, 1995–2018. **A** 10-year age-standardized relative survival for 1995–2018; **B** 5-year age-standardized relative survival for 1995–2006 and 2007–2018. The dashed lines indicate 95% confidence intervals and insert of each graph displays the same curve with a magnified *y*-axis.

In complete analysis (including the entire follow-up in 1995‒2018), the 10-year age-standardized relative survival was 94% (95% CI, 89%‒100%) (Fig. 3A). In period analysis, the 5-year age-standardized relative survival was 96% (95% CI, 92%‒100%) in 2007‒2018 compared with 89% (95% CI, 83%‒95%) in 1995‒2006 (Fig. 3B).

Adjusted HRs for excess mortality are shown in Table 2. Older age at diagnosis was associated with increased excess mortality (HR, 1.75 per 10-year increase; 95% CI 1.15‒2.66; *p* = 0.009), whereas patients diagnosed at a later calendar year had lower excess risk of death (HR, 0.38 per 10-year increase; 95% CI, 0.15‒0.98; *p* = 0.046). There was no significant sex difference in excess mortality. Transformation was associated with a substantial increase in excess mortality (HR, 26.7; 95% CI, 9.41‒75.9; *p* < 0.001), but the excess relative risk appeared to decrease over time. The time-dependent excess HRs at 1, 5, and 10 years after transformation were 30.7 (95% CI, 12.2‒77.1), 6.79 (95% CI, 1.16‒39.6), and 7.39 (95% CI, 1.18‒46.1), respectively.

### Causes of death

Of the 78 deaths during the study period, 32 (41%) were due to any lymphoma, 14 (18%) to secondary malignant neoplasm, and 32 (41%) to other causes. Other causes of death were most frequently related to cardiovascular and infectious diseases. Of the 12 deaths recorded in the patients with incident transformation, 11 (92%) were due to any lymphoma.

## Discussion

In this large nationwide, population-based cohort study of the patients diagnosed with NLPHL in Finland between 1995 and 2018, the risk of transformation was 6.3% at 10 years, resulting in a substantially increased mortality in comparison to the patients without transformation. Overall, however, we found that the 10-year relative survival for the entire study period was excellent. Our results also suggest a reduction in excess mortality over time and a slight improvement in 5-year relative survival between 1995-2006 and 2007-2018. Finally, excess mortality was higher for the patients diagnosed at older ages.

The incidence of NLPHL has nearly doubled in some countries since the 1990s, reaching 2-3/1,000,000 during the 2000s [5, 15, 31]. Whether this represents a true rise or evolving diagnostic practice is unclear. In contrast, the incidence of NLPHL in Finland has remained stable at 3/1,000,000 since the 1990s [7]. One possible explanation may lie in the diagnostic centralization of rare lymphomas to university hospitals and review of most cases by hematopathologists in Finland, which is known to increase diagnostic accuracy [32, 33]. In this study, 80% of cases were diagnosed in one of five university hospitals or reviewed by an expert hematopathologist.

The high proportion of males (76%) in our cohort is consistent with previous studies [8, 15]. The median age of 48 years at diagnosis is also in line with a population-based study from Sweden [34]. In addition, we observed that females were significantly older than males at the time of diagnosis. Interestingly, a study from the United States found approximately equal proportions of females and males among African American patients diagnosed with NLPHL [35]. Furthermore, Caucasian females were more likely to be diagnosed at a later age than African American females. However, a direct comparison with our study is not feasible considering differences in ethnical background between the Finnish and the United States population.

In general, NLPHL is associated with excellent prognosis and low excess mortality [15, 17, 34, 36]. In the present study, the 10-year age-standardized relative survival was 94%, which is consistent with a recently published population-based study from the Netherlands [15]. In addition, 5-year relative survival improved slightly between 1995−2006 and 2007−2018. Deaths during the study period were attributed to any lymphoma (41%), secondary malignant neoplasms (18%), and other causes (41%). Lymphoma was a major cause of death in patients with incident transformation (92%).

The risk of transformation was 6.3% (95% CI, 4.2–9.6) at 10 years, which is lower than in two other relatively large studies [12, 13]. In a French registry-based study, the 10-year cumulative risk of transformation, based on the Kaplan-Meier method, was 12% (95% CI, 7‒17) with a high biopsy rate at recurrence (85%) [12]. In a British study, transformation occurred in 26 of 153 patients (17%), but the risk was not estimated for a specified time interval [13]. The majority of the previous studies have been limited either by lack of population-based data collection (single-institution or pooled multi-center studies), small sample size, short follow-up time, lack of histological verification of transformation, or inclusion of composite lymphomas [10, 11, 14, 37–40].

We excluded 16 patients with composite lymphoma from the study. It is, however, possible that these patients had undiagnosed NLPHL, which eventually transformed into composite lymphoma. On the other hand, composite lymphoma may have developed as a primary neoplasm.

Our results indicated that the risk of death was substantially higher in patients with transformation compared with patients without transformation. Considering transformation as a time-varying covariate avoids immortal time bias, which is a common problem in the medical literature [41]. A possible explanation for the decrease in relative risk over time is that patients who have survived a given length of time after transformation represent a selected population with less aggressive disease, better treatment response, or otherwise more favorable prognostic factors than the complete population of patients with transformation.

Increased age at diagnosis was associated with higher excess mortality. The use of general population mortality rates as a reference allows the study of age at diagnosis as a predictor of disease- or treatment-related mortality, whereas total mortality is strongly influenced by causes unrelated to NLPHL. Treatment toxicities may be more frequent among elderly patients, but the biology of NLPHL could also differ between age groups. There was no significant association between sex and excess mortality, which is in accordance with a large study from the Netherlands [15]. However, future population-based studies are warranted to validate this finding.

Interestingly, we found a significant reduction in excess mortality for the patients diagnosed with NLPHL during more recent years. We hypothesize that mortality may have decreased gradually over time due to a shift toward less aggressive treatment approaches both in up-front and recurrence settings, including a higher proportion of patients on active surveillance, the use of less extended radiation fields, and the introduction of biological agents into treatment regimens. In a population-based study from Sweden, the use of rituximab increased from 7% to 67% between 2000–2004 and 2005–2014 in the first line treatment for advanced-stage NLPHL [34]. We expect that treatment trends in Finland are similar to the other Nordic countries and follow international guidelines [19]. Furthermore, higher biopsy rates at recurrence in more recent years could have resulted in better detection of incident transformations with adequate treatment given accordingly. However, the risk of transformation was not associated with the period of diagnosis in our study. Taken together, these factors may have reduced both early and late treatment-related mortality over time. Overdiagnosis is unlikely to explain the decrease in excess mortality because the incidence of NLPHL remained stable during the study period.

The strengths of this study are the population-based design and the use of high-quality nationwide cancer registry data from a long calendar period of 24 years. Furthermore, all NLPHL diagnoses and transformations were reviewed from the original pathology reports at the Finnish Cancer Registry, and 80% of the cases were diagnosed in one of Finland’s five university hospitals or reviewed by an expert hematopathologist.

However, some limitations require careful consideration. First, we could not perform a central pathology review to confirm the diagnosis of NLPHL. Second, detailed individual-level data on treatment was unavailable. Although the patients with NLHPL in Finland are treated according to international guidelines, which strongly recommend a biopsy confirmation prior to establishing a diagnosis of histologic transformation, we cannot exclude the possibility that some patients were treated for presumed transformation based solely on clinical findings. Third, the FCR did not have information on stage, performance status, or comorbidities.

Collectively, our data underscore the importance of long-term follow-up, re-biopsy at the time of relapse, and minimization of the risks for late adverse effects associated with treatment.

In conclusion, this large population-based cohort study with long-term follow-up demonstrates excellent long-term relative survival for patients diagnosed with NLPHL in Finland between 1995 and 2018. Excess mortality was lower among NLPHL patients diagnosed in more recent years. The risk of transformation to aggressive B-cell lymphoma was 6.3% at 10 years, and transformation was associated with a substantially increased risk of death.

## Supplementary information

Reproducibility checklist
